# Anticipatory action planning for stepping onto competing potential targets

**DOI:** 10.3389/fnhum.2022.875249

**Published:** 2022-08-22

**Authors:** Ryo Watanabe, Takahiro Higuchi

**Affiliations:** ^1^Department of Health Promotion Sciences, Tokyo Metropolitan University, Tokyo, Japan; ^2^Research Fellow, Japan Society for the Promotion of Science, Tokyo, Japan

**Keywords:** anticipatory action planning, postural stability, stepping, sensorimotor control, go-before-you-know

## Abstract

The brain plans an anticipatory action for performing tasks successfully and effortlessly even if there are multiple possible options. There is increasing evidence that, when multiple actions are possible, the brain considers two factors when planning an anticipatory action—the probabilistic value and the action cost for each potential action. When the action involves maintaining upright balance, such as standing, stepping, or walking, the action cost for maintaining postural stability could be considered dominantly. We addressed this issue by using a “go-before-you-know” task to step onto a target on the floor. In this task, two potential targets were located on the medial or lateral side of the stepping foot, and the true target was cued only after participants shifted their loads to leave that foot. Participants initiated their stepping actions without knowing which of the potential targets would be the true one. The results showed that, for the majority of participants, lateral displacements of the center of pressure (COP) with two potential targets were similar to those when a single target exists on the individual’s medial side. Given that mediolateral postural stability became more destabilized with stepping onto the medial target than stepping onto the lateral target, they were likely to plan their mediolateral components of the postural adjustments for the worst-case scenario (i.e., falling). Additionally, posterior COP movements with two potential targets became smaller than those with a single target, suggesting an effort to create extra time to determine the true target and to adjust the swing foot. Based on these findings, we concluded that action costs for maintaining postural stability were considered dominantly for planning an anticipatory action to accomplish a stepping task successfully while ensuring upright balance.

## Introduction

An individual prepares to take a certain action by considering multiple possible options (e.g., walking while preparing for stepping on his/her left and right sides in response to the walking direction of a pedestrian coming toward him/her). Previous studies have indicated that, when planning an anticipatory action, the brain considers at least two factors regarding a situation—the probabilistic value and the action cost for each potential option (Christopoulos and Schrater, 2015; Enachescu et al., 2021). When individuals are standing, stepping, or walking so that maintaining postural stabilities is a major issue for control, it is possible that the action cost for maintaining their postural stability would be considered dominantly for planning an anticipatory action. The purpose of the present study was to investigate whether our idea would be the case.

The first factor considered for anticipatory action planning is the probabilistic value. Typical studies investigating anticipatory action planning have used a reaching task in which two potential targets were presented simultaneously prior to initiating reaching action (a go-before-you-know task). In this task, participants initiated their reaching before knowing which one would be cued as the target to reach (the true target). When both targets could be the true target with the same probability, the initial reach was aimed toward an intermediate location between both targets (Chapman et al., 2010). When one of two targets was selected to be the true target more frequently, the initial reaching trajectory tended to be biased toward that target (Enachescu et al., 2021). The trajectory was not a straight path toward the likely target but rather an average of trajectories toward potential targets weighted by the target probability. Such a tendency has been referred to as “motor averaging.” One plausible explanation for motor averaging is that the sensorimotor system specifies multiple actions for each potential target in parallel and then plans an average action of them weighted by the probabilistic values (Chapman et al., 2010). Motor averaging behaviors are also observed in the reaching task when more than two possible targets appear (Gallivan et al., 2011; Gallivan and Chapman, 2014).

Another factor considered is the action cost required to perform an action. Nashed et al. (2017) used a modified version of the go-before-you-know task (Nashed et al., 2017). In their task, participants reached toward two potential targets while grasping the manipulandum. The elastic load of the manipulandum was manipulated with a robotic device. Large elastic loads from each target toward the midline of both targets were delivered to the manipulandum when reaching toward each target. In this condition, minimal loads were sufficient to initiate moving the manipulandum. However, if the upcoming initial action is planned based on the averaging strategy, which means that actions toward the left and right targets are planned in parallel and averaged, then the initial grip force should become greater in an effort to resist the elevation of the elastic load as the manipulandum moved closer to each target. However, the result showed that the grip force was scaled for the minimal load. This suggests that it is the single movement plan to minimize effort, rather than the averaging of the potential actions, that was considered in planning the reaching behavior.

When individuals stand, step, or walk, maintaining stability (i.e., not falling) is exclusively dominant. Even if stepping onto a target is preferable, individuals would not select stepping on that target when it causes destabilization that would lead to a fall. In this case, it is possible that the brain considers action costs for maintaining postural stabilities more dominantly than other factors. When stepping onto a certain location, a landing location with respect to the center of mass (COM) of the whole body is one factor affecting postural stability (Moraes, 2014; Bruijn and van Dieën, 2018). Previous studies have shown that mediolateral postural stability became more destabilized when correcting the foot placement toward the medial side than toward the lateral side (Moraes et al., 2007; Sun et al., 2017). This is because an adjustment in the medial direction narrows the base of support (BOS), which is the area within an outline of all points formed by feet that are in contact with the floor at any point in time (Bruijn and van Dieën, 2018). Accordingly, Reynolds and Day (2005) demonstrated that, when individuals must step and maintain stability in response to a sudden change in landing location, the magnitudes of corrections were less when the swing foot was corrected toward the medial side than toward the lateral side. Importantly, this tendency was more evident when performing the task without any balance support, such as holding handrails (Reynolds and Day, 2005). These findings suggest that stepping actions are planned in consideration of the potential threat to balance when correcting the foot toward the medial side.

Based on these findings, we hypothesized that, when an action involves maintaining upright balance, the action cost for maintaining postural stability is predominantly considered in planning an anticipatory action to accomplish a task successfully. Even if two potential targets for stepping are located on the medial and lateral side of the stepping foot and either is selected as the true target with the same occurrence rate, the initial stepping action would be planned to step onto the medial target. This was expected because mediolateral postural stability became more destabilized when correcting the foot placement toward the medial side than toward the lateral side (Moraes et al., 2007; Sun et al., 2017).

To test this, we used a go-before-you-know task to step onto a target on the floor. Previous studies using a single-stepping task revealed that postural adjustments occurred during the pre-step phase, i.e., the quiet-stance phase, which lasts until the moment of lifting the stepping foot, were strictly controlled to step onto an intended location (Lyon and Day, 1997, 2005; Bancroft and Day, 2016). Several studies using a gait-initiation task showed the close relationship between postural adjustments during the pre-step phase and spatial accuracy of the landing location (Corbeil and Anaka, 2011; Yiou et al., 2016). Based on these studies, we focused on the anticipatory postural adjustments observed during the pre-step phase to determine whether individuals intend to plan for an action to effectively avoid upcoming postural disturbances. More specifically, we focused on lateral displacements of the center of pressure (COP) toward the swing-foot side, which precedes the lift of the stepping foot. For example, prior to stepping toward the individual medial side, the COP is displaced more toward the swing-foot side (Corbeil and Anaka, 2011). If action costs for maintaining postural stability are negligibly small for a planned action, the magnitudes of the lateral COP displacements with two potential targets were similar to the intermediate magnitude between those prior to stepping actions on the medial and lateral target. However, if these costs are taken into account dominantly, then the magnitudes of the lateral COP displacements would be similar to those when a single stepping target is on the individual’s medial side.

A previous study also showed that individuals tend to move slowly to compensate as target uncertainty increases (Hudson et al., 2007). Such an adjustment could be beneficial, given that it creates extra time for determining the true target. If the brain follows the same rule for planning a stepping action, postural adjustments with multiple potential targets may also be organized to decelerate the body and afford time for decisions and movement corrections. Posterior displacements of the COP before raising the stepping foot off the ground are important for altering the velocity of the body for forward progression (Corbeil and Anaka, 2011; le Mouel and Brette, 2017). We expected that posterior displacements of the COP with two potential targets would be smaller than those with a single target to creates extra time for determining the true target and adjust the swing foot. Based on the results of testing these hypotheses, we discussed whether emphasis would be placed on the action cost for maintaining postural stability when the action involves maintaining upright balance.

## Materials and methods

### Participants

Fourteen young individuals (six females) participated in this experiment. All participants were right-leg dominant and had not reported any history of musculoskeletal or neurological disorders in their self-reports. This experiment was approved by the Ethics Committee of Tokyo Metropolitan University (approval number: H2-65). All participants provided written informed consent and received a bookstore gift card for their participation. The data obtained from one participant was excluded from the following analysis due to system failure. We used data obtained from 13 participants for the following analysis procedure (age: 23.0 ± 3.4 years; height: 164.3 ± 11.3 cm; weight: 60.0 ± 10.2 kg).

### Apparatus

**FIGURE 1:**
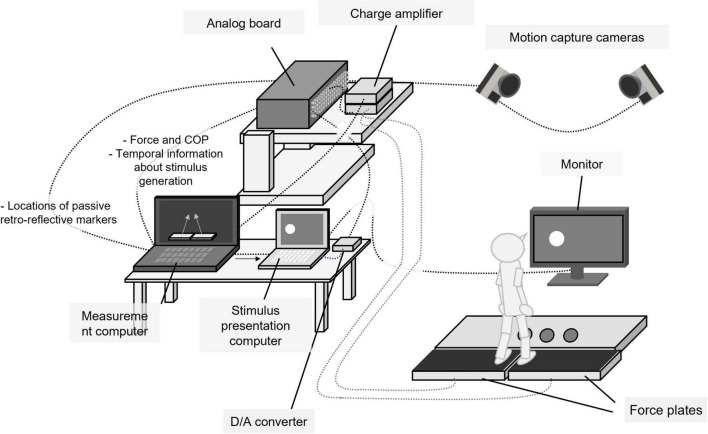
Experimental setup.

The experimental setup is shown in Figure 1. It consisted of two computers for data measurement and stimulus presentation, a 27-inch LCD monitor with 60 Hz (LCD-MF276XD, I/O DATA, Japan), 14 cameras for three-dimensional motion capture (Oqus300SYS, Qualisys, Sweden), two force plates (Kistler 9286AA type and 9286BA type, Kistler, Switzerland), an analog board (64-channel analog interface, Qualisys, Sweden), and a D/A converter (MMB Trigger Box, Neurospec, Switzerland). Fourteen passive retro-reflective markers were attached to seven anatomical landmarks of each participant’s lower body bilaterally (second toe top, first metatarsal, second metatarsal, fifth metatarsal, heel, anterior superior iliac spine, and posterior superior iliac spine). Spatial locations of markers were tracked with three-dimensional motion cameras at a sampling frequency of 100 Hz and processed with the motion capture software (Qualisys Track Manager, QTM; Qualisys, Sweden). QTM was also used to integrate and synchronize all data obtained in the experiment. The ground reaction forces and COP were measured with the two force plates at a sampling frequency of 1,000 Hz. These data were recorded with the QTM through a 64-channel analog board. Software (TRIAS2, Q’sfix, Japan) was used to control a charge amplifier of both force plates and initialize the states of plates before each trial started.

Visual and auditory stimuli were generated using PsychoPy3 (Peirce et al., 2019). All visual stimuli were displayed on the monitor. Temporal information about stimulus generation was sent to the QTM as analog signals through a D/A converter (MMB Trigger Box, Neurospec, Switzerland). To calculate a participant’s load shift during each trial in real time and display visual stimuli with his/her movements, the force data were sent from the QTM to a customized Python program (Qualisys Python SDK, Qualisys, Sweden), and the differences between vertical forces acquired from both force plates were compared using the measurement computer. A signal was sent from the measurement computer to the stimulus-presentation computer to change the visual stimuli when the difference between both vertical forces exceeded the setting values (10% of total body weight). There were processing several delays from the sending of the signal to the changing of the visual stimuli on the monitor. Preliminary measurement using a high-speed camera sampling frequency of 240 Hz showed that the delay was estimated to be about 217 ms.

### Task and protocol

**FIGURE 2:**
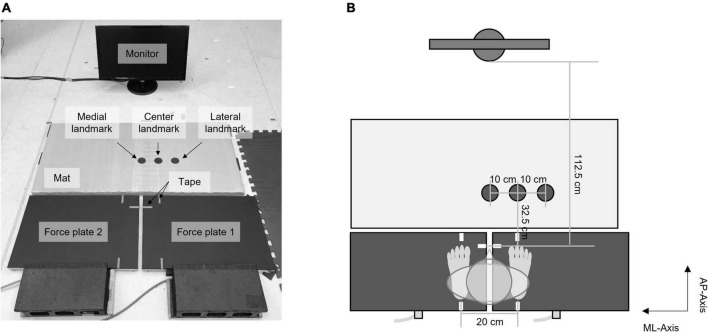
Task setup: **(A)** a picture of the task setup; **(B)** top view of the configuration of the task setup.

The task setup is shown in Figure 2. Three circle landmarks were located on an ethylene-vinyl acetate mat 32.5 cm in front of the participant’s toe position (Figure 2A). The central landmark was located ahead of the right foot (i.e., the swing foot), whereas the lateral and medial landmarks were located 10 cm away from the center landmark. The monitor was located on the floor 112.5 cm in front of the participant.

Participants stood barefoot on the dual force plates. They were instructed to adjust their toes and heels to correspond with tapes on each force plate so that, at the start of each trial, the stance width was maintained at 20 cm, and the anterior–posterior distance between the target and the right foot was 32.5 cm. Participants also tried to distribute their loads evenly between both feet.

**FIGURE 3:**
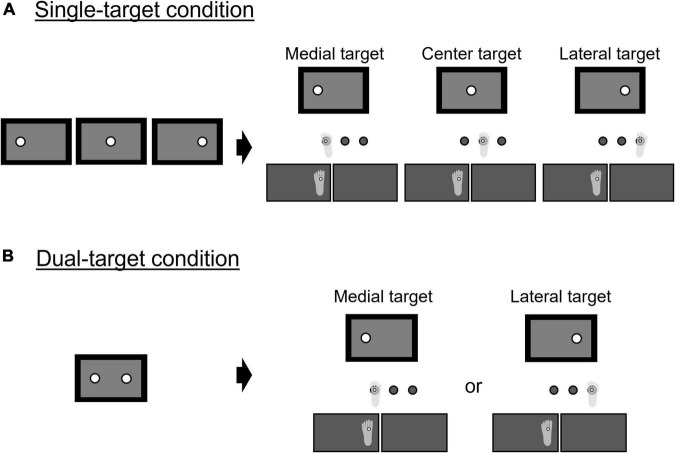
Illustration of trial types: **(A)** single-target condition; **(B)** dual-target condition.

Each trial started with a plus-shaped fixation point presented on the center of the monitor for 1,000 ms. As soon as the fixation point disappeared, the first auditory beep was presented concurrently with either one or two targets shown on the monitor. In the single-target condition (Figure 3A), a single target appeared on either the center, right, or left side of the monitor with the first auditory beep. After a random interval (1,000–1,500 ms) after the target’s appearance, a secondary auditory beep cued participants to step onto the floor landmark corresponding to the target location presented on the monitor (see Figure 3A). There was no time limit for generating an action after a go signal. However, participants were instructed that they should try to step quickly and accurately on the specified landmark so that the marker attached to their second metatarsal bone head of the swing leg would align vertically with the center of the circle. In the dual-target condition (Figure 3B), two potential targets were presented simultaneously on the left side and the right side of the monitor. The true target was selected with the same occurrence rate from both targets (medial: lateral = 1: 1). After a random interval (1,000–1,500 ms), a second auditory beep cued participants to start moving while they did not know which was the correct target. The true target was displayed, while the other potential target disappeared when the difference in vertical force between the right- and left-foot sides exceeded 10% of the total body weight. The threshold value of 10% was determined based on our pilot study. It was ideal to present the true target as soon as the peak of the lateral displacements of the COP on the swing side occurred (i.e., it was presented at the timing between the unloading and early swing phase). If the true target was presented much earlier than that, participants could adjust their COP movements corresponding to the location of the true target. If it was much later (i.e., it was presented during the mid-swing phase), then participants could not correct the swing foot to the true target sufficiently (Young and Hollands, 2012). Because there was a mechanical delay of about 217 ms between the input of the force values and the output presenting the true target, we needed to explore the timing to reliably present the true target around the peak of the lateral displacement of the COP. In the pilot study, we found that setting the threshold value at 10% of the total body weight was reasonable for dealing with the issue.

Trials in which participants loaded unevenly on either the right or left foot over threshold values at the moment of the second auditory beep were regarded as invalid and were not included in the main trials. In such trials, either “R OVER” or “L OVER” was displayed on the monitor just after the secondary auditory beep to ask participants to avoid standing unevenly.

Participants performed a total of 240 main trials. Trials were divided into sets of 120 trials per day to avoid fatigue. Each day, participants performed the task for 72 trials in the single-target condition and 48 dual-target trials. In the single-target condition, each of the center, right, and left targets appeared for 24 trials. In the dual-target condition, each of the right and left targets was selected as the true target for 24 trials. Trials regarded as invalid for uneven loads were subtracted from the trials of each condition. The trial order of the single-target trials and dual-target trials was randomly intermixed, which is the same as in the previous study (Wong and Haith, 2017). To avoid fatigue, participants rested for more than 2 min in every 30 trials. All participants completed each day’s tasks within 3 h.

To familiarize participants with the task, they performed 20 training trials on both days before the main trials. In this session, participants first completed 10 trials in which two trials for all target conditions of both single-target trials and dual-target trials were performed in the following order sequentially: single-center, single-lateral, single-medial, dual-lateral, and dual-medial. Participants completed 10 trials, in which two trials for each target condition were presented in randomized order.

### Data analyses

Before data processing, all force plate data and all marker data were offline low-pass filtered at 20 and 4 Hz, respectively (fourth-order Butterworth). The global COP position was calculated from the output of both force plates according to the following equation (Honeine et al., 2016):


$${\text{COP}}_{g\,l\,o\,b\,a\,l}=\left[\left({\text{Fz}}_{1}*{\mathrm{COP}}_{1}\right)+\,\left({\text{Fz}}_{2}*{\mathrm{COP}}_{2}\right)\right]/\,\left({\text{Fz}}_{1}+{\text{Fz}}_{2}\right),$$

where Fz_1_ and Fz_2_ are the vertical ground reaction forces on the left- and right-foot sides, respectively, and COP_1_ and COP_2_ are the COP positions on the left- and right-foot sides, respectively. The coordination system of COP_1_, COP_2_, and global COP were based on the global coordinate system. The velocity of global COP was calculated by time derivatives using the three-order central difference method.

The onset of the lateral COP shift was determined as the first point at which the medial-lateral velocity of the global COP toward either foot side exceeded 0.05 m/s and then continued for at least 50 ms (Bancroft and Day, 2016). The lateral peak point of the COP movement was defined as the mediolateral peak of the COP movement toward the swing (right) foot, as established in the previous study (MacKinnon et al., 2007; Corbeil and Anaka, 2011). The posterior peak point of the COP movement was also defined as the posterior peak of the COP movement between the onset of the lateral COP shift and the posterior peak point of the COP movement. Notably, multiple peaks of COP movements were observed in some trials. Considering this issue, and to avoid contamination of feedback adjustments in response to the true target presentation, we defined the initial peak of these COP patterns as the lateral and posterior peak point of the COM movement. The lateral displacement of the COP toward the swing limb was defined as the lateral movement distance of the COP from the position at the onset of the COP movements to the position at the initial lateral peak of the COP movements (Corbeil and Anaka, 2011). The posterior displacement of the COP toward the swing limb was defined as the posterior movement distance of the COP from the position at the onset of the COP movements to the position at the initial posterior peak of the COP movements. To evaluate dynamic postural stabilities when landing the swing foot on the target, the margin of stability (MOS) at foot contact was calculated. The MOS was defined as the distance between the boundary of the BOS and the extrapolated COM (XCOM) at foot contact. Foot contact was determined as the first point at which the vertical velocity of the second metatarsal marker exceeded −0.02 m/s from the minimum point. We calculated the MOS by using pelvis markers and foot markers following the definition of Sun et al. (2017). The location of the COM was estimated from the average position of the anterior superior iliac spine (ASIS) and posterior superior iliac spine (PSIS) markers. The XCOM was calculated according to the following equation (Hof et al., 2005):


$$X\,C\,O\,M=C\,O\,M+v/\sqrt{g/L},$$

where *v* is the COM velocity, g is the gravitational acceleration (9.81 m/s^2^), and *L* is the vertical distance between the COM and the average position of the right and left heel marker during quiet stance. The MOS in the anteroposterior direction (MOS_AP_) was defined as the distance between the toe marker of the swing foot and the XCOM. The MOS in the mediolateral direction (MOS_ML_) was defined as the distance between the fifth metatarsal marker of the swing foot and the XCOM. The movement time was determined as the time from the onset of the COP to the swing foot’s contact. These analyses were performed using a customized program in MATLAB (MATLAB ver. R2020a, MathWorks, United States, Natick, MA, United States).

### Statistical analyses

Before statistical analyses, we excluded the following trials: (1) between the first auditory sound and the second auditory sound, participants stood with their weight uneven (over 55% of their weight on either side); (2) the onset of the COP movement was detected before the second auditory sound; (3) the COP shifted initially toward the stance limb side and/or the forward direction just after the onset of the COP, which meant an abnormal COP pattern; (4) the true target was presented before the timing at the initial lateral or posterior peak of the COP movements; (5) multiple steps were required to stop after stepping onto a target. The number of datasets used for the statistical analyses are shown in Supplementary Table 1. We realized that there was little valid data in the dual-target condition of participant ID2 as compared with that of other participants. We checked whether including or excluding data of participant ID2 affected the following statistical procedures. The results showed that the rejection of null hypotheses in statistical analyses remained unchanged, regardless of whether the data of participant ID2 was included or not. Therefore, we included the data of ID2 to avoid a smaller sample size.

The main dependent variables were the lateral displacement of the COP toward the swing limb, the posterior displacement of the COP toward the swing limb, the MOS_ML_, at foot lift, the MOS_AP_ at foot lift, and the movement time. Regarding the learning effect of performing the task, we performed a preliminary analysis using a three-way (day, number of targets, and stepping side) analysis of variance (ANOVA) with repeated measures. We confirmed whether there were any learning effects according to a main effect or interactions between the day and other factors. Afterward, the data of day 1 and day 2 were combined and averaged to focus on the effects of the number of targets and the stepping side. As the main analysis, a two-way (number of targets and stepping side) ANOVA with repeated measures was performed. The threshold of significance was set at *p* < 0.05. Effect sizes are reported as partial η*^2^* ($\eta_{\text{p}}^{2}$) statistics for the relevant main and interaction effects. Greenhouse–Geisser corrections were applied to the degrees of freedom if violations of the assumption of sphericity were detected in Mendoza’s multisample sphericity test. Statistical procedures of ANOVA were performed using the *anovakun* function (ver. 4.8.5) in R.

According to the patterns of lateral COP displacement in the dual-target condition, we additionally performed a one-sample *t*-test and a power analysis to determine whether the magnitudes of lateral COP displacements in the dual-target condition were more like those in the single-medial condition than the intermediate value between those in the medial and lateral conditions. For this, we normalized the COP displacement data for each participant. First, lateral displacements of the COP pooled from both the dual-lateral and dual-medial target condition were subtracted from the intermediate value between the means of the single-lateral and single-medial conditions and then divided by the deviation from the intermediate value between the means in both single conditions to the mean of the single-medial condition. After applying these transformations, we compared the mean of the normalized magnitudes of lateral COP displacements in the dual-target condition with the intermediate value between the means of both single conditions (setting a population mean = 0) or the mean of the single-medial condition (setting a population mean = 1), respectively. Additionally, a statistical power was calculated using the mean of normalized magnitudes of lateral COP displacements in the dual-target condition of this data. Statistical procedures of a one-sample t test and a power analysis were performed using the *t.test* function and the *pwr.t.test* function in R, respectively.

We also performed hierarchical Bayesian modeling to reveal the individual weight between the policy of medial stepping and lateral stepping. Specifically, using the data of the lateral displacements of the COP, we estimate the parameters: weighting values θ_(*k*)_, which were fitted to each participant (*k*), and θ, which was a common parameter across all participants. Analyzing the data using the Bayesian modeling method makes possible more stable estimations of participant-specific parameters with small sample sizes by using all data of participants and partially using individual-level data.

In detail, we set the model structure as follows:


$$\,\alpha_{\left(k\right)}∼N\,o\,r\,m\,a\,l\,\left(\alpha ,\sigma_{\alpha }\right),$$

$$\,\theta_{\left(k\right)}=i\,n\,v\,e\,r\,s\,e\,l\,o\,g\,i\,t\,\left(\alpha_{\left(k\right)}\right),$$

$${\bar{\mu }}_{D\,\left(k\right)}=\theta_{\left(k\right)}*{\bar{\mu }}_{S\,M\,\left(k\right)}+\left(1-\theta_{k}\right)*{\bar{\mu }}_{S\,L\,\left(k\right)},$$

$$\,Y_{\left(k,i\right)}∼N\,o\,r\,m\,a\,l\,\left({\bar{\mu }}_{D\,\left(k\right)},\sigma_{\left(k\right)}\right).$$

We set α as the group-level parameter to identify a dominant action policy among all participants. Additionally, we set σ_*a*_ to consider individual differences in action policies. Therefore, *a*_*k*_ represents the individual parameter according to a Gaussian distribution (mean = α, sigma = σ_α_). α_*(k)*_ was converted into θ_*(k)*_, which has the interval [0, 1], by using an inverse-logit transformation. θ_*(k)*_ represents the individual weight between the policy of medial stepping and lateral stepping. ${\bar{\mu }}_{S\,M\,\left(k\right)}$ and ${\bar{\mu }}_{S\,L\,\left(k\right)}$ are the average values acquired from lateral stepping and medial stepping in the single-target condition, respectively, for each participant. ${\bar{\mu }}_{D\,\left(k\right)}$ is the weighted average under both ${\bar{\mu }}_{S\,M\,\left(k\right)}$ and ${\bar{\mu }}_{S\,L\,\left(k\right)}$, which are weighted with the parameter θ_*(k)*_. σ_*(k)*_ represents the standard deviation of the Gaussian function for fitting the pooled data from each participant in this study. We fitted the Gaussian function with the mean ${\bar{\mu }}_{D\,\left(k\right)}$ and standard deviation σ_(*k*)_ to the data of lateral displacements of the COP pooled over the lateral target condition and medial target condition in the dual-target condition *Y*_*(k,      i)*_. At group-level policy parameter α, we selected a weak prior using a Gaussian distribution (mean = 0, sigma = 10000). At the policy variance σ_*a*_, we selected a weak prior using the half-Cauchy distribution (mean = 0, scale = 25). These weak priors were selected independently of the data based on a previous study using hierarchical Bayesian modeling (Gelman, 2006). At the individual level of variance among dual-target trials σ_(*k*)_, we specified a prior parameter using a uniform distribution (lower = 0, upper = 1,000). This prior parameter was selected for having an equal probability across a wide range of positive values. Posteriors were calculated using Markov chain Monte Carlo sampling based on the Hamiltonian Monte Carlo method. The sampling method was based on the No-U-Turn Sampler (NUTS) algorithm. We produced four chains with 25,000 samples. Simulations were preceded by 5,000 burn-in steps, which were excluded due to the collection of samples from a stationary distribution, and the remaining 20,000 were used for each parameter estimation. Convergence checking was executed based on R-hat diagnostic values, which were below 1.1 among all parameters. All procedures of our Bayesian modeling and output of results were performed using Rtools (ver. 4.0) and the Rstan package (ver. 2.21.2) in R (ver. 4.1.0).

To reveal the relationship between the COP displacements and the movement time, we performed a trial-by-trial analysis. A dataset of posterior COP displacements and movement times was pooled from each trial in the single-lateral, single-medial, dual-lateral, and dual-medial condition. We fitted a linear regression model to the dataset for each participant. Statistical procedures of a linear regression were performed using the *lm* function in Python.

## Results

### Preliminary analyses for testing the possibility of learning

Because the data were collected separately for 2 days (120 trials, including 48 dual-target trials per day), there was a possibility of showing better performance on the second day than on the first (i.e., the learning effect). Therefore, we conducted a preliminary analysis to test the possible learning effect. We used a three-way repeated ANOVA with repeated measures of the day, number of targets, and stepping side for all dependent variables. The results showed that for all except movement time, neither the main effect of the day nor interactions including the day were found, suggesting that there was no learning effect. For the movement time, the main effect of the day [*F*_(1,12)_ = 11.29, *p* = 0.006, $\eta_{\text{p}}^{2}$ = 0.48] and its interaction with the stepping side [*F*_(1,12)_ = 4.87, *p* = 0.048, *$\eta_{\text{p}}^{2}$* = 0.29] were significant, suggesting that participants performed a stepping task more quickly on the second day than on the first in both medial and lateral target conditions. Based on preliminary analyses, we concluded that the learning effects were relatively low.

### Comparing the magnitudes of the lateral displacements of the center of pressure toward the swing leg between single- and dual-target conditions

**FIGURE 4:**
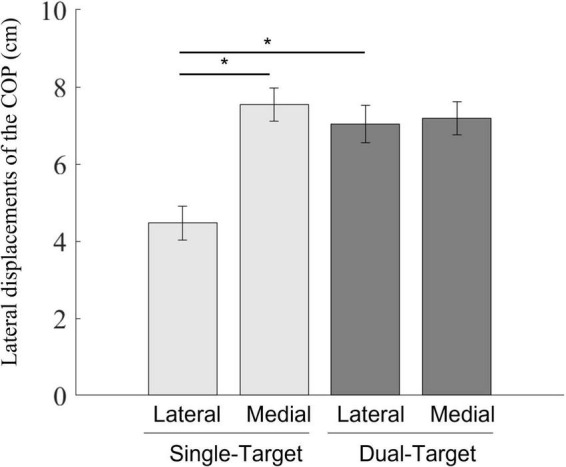
Mean lateral displacements of the COP between the position at the onset of the COP movements and the position at the initial lateral peak of the COP movements toward the swing foot side. Light gray bars represent average values in the single-target condition, and dark gray bars represent average values in the dual-target condition. Error bars represent standard errors of the mean. An asterisk (*) indicates a significant difference in group means based on a *post hoc* analysis (*p* < 0.05).

The result figure is shown in Figure 4. A two-way repeated-measures ANOVA showed significant main effects of the number of targets [*F*_(1,12)_ = 26.33, *p* < 0.001, *$\eta_{\text{p}}^{2}$* = 0.69] and the stepping side [*F*_(1,12)_ = 32.66, *p* < 0.001, $\eta_{\text{p}}^{2}$ = 0.73]. Lateral displacements of the COP were larger in the dual condition than those in the single condition. As a factor of stepping sides, lateral displacements of the COP were larger in the medial condition than those in the lateral condition. The interaction was also significant [*F*_(1,12)_ = 71.62, *p* < 0.001, $\eta_{\text{p}}^{2}$ = 0.86]. Simple main effects of the interaction between two factors revealed that lateral displacements of the COP were larger in the dual-lateral condition than those in the single-lateral condition [*F*_(1,12)_ = 60.16, *p* < 0.001, $\eta_{\text{p}}^{2}$ = 0.83]. In addition, lateral displacements of the COP in the single-medial condition were larger than those in the single-lateral condition [*F*_(1,6)_ = 69.01, *p* < 0.001, $\eta_{\text{p}}^{2}$ = 0.85]. There was no significant difference between the single-medial condition and the dual-medial condition [*F*_(1,12)_ = 2.95, *p* = 0.112, *$\eta_{\text{p}}^{2}$* = 0.20]. There was also no significant difference between the dual-medial target condition and the dual-lateral target condition [*F*_(1,12)_ < 1.0, *p* = 0.598, $\eta_{\text{p}}^{2}$ = 0.02].

A one-sample *t*-test and a *post hoc* power analysis were performed to address whether the mean of the normalized magnitudes of lateral COP displacements in the dual-target condition would be similar to those in the single-medial condition (Supplementary Figure 1). Normalized magnitudes of the lateral COP displacements in the dual-target condition were significantly larger than the intermediate value between the means of those in the single-lateral and single-medial conditions [*t*_(12)_ = 4.94, *p* < 0.001, *d* = 1.37, *power* = 0.99], whereas normalized magnitudes of the lateral COP displacements in the dual-target condition were not significantly smaller than those in the single-medial condition [*t*_(12)_ = −1.57, *p* = 0.142, *d* = 0.44, *power* = 0.31]. In summary, lateral displacements of the COP in the dual-target condition were comparable to those of stepping toward the medial side in the single-target condition.

### Hierarchical Bayesian estimation of individual weight between the policies of medial stepping and lateral stepping

**FIGURE 5:**
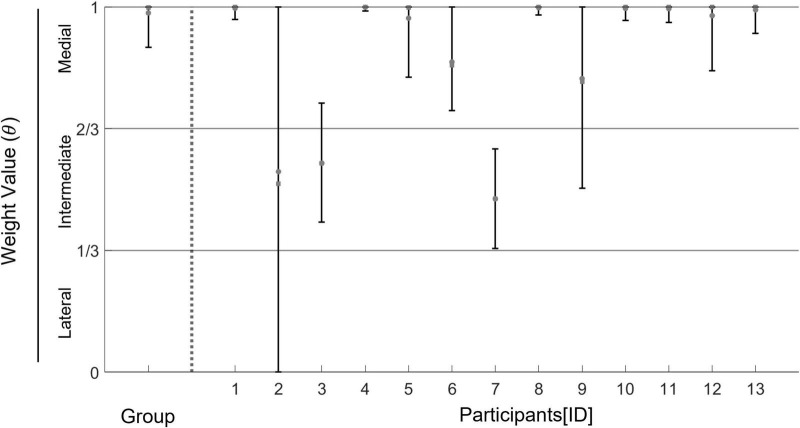
Estimated values of a group weight and individual weights between the policies of medial stepping and lateral stepping estimated in the dual-target condition. Round dots represent a posterior mean of the value of weight between the medial stepping and the lateral stepping for each participant. Square dots represent a posterior median of the same parameter for each participant. Error bars represent a 95% creditable interval of each estimated parameter. Horizontal lines represent 1/3 and 2/3, respectively. These lines are the interest value for judging whether the COP pattern for each participant is either a “medial weighing pattern”, a “lateral weighing pattern”, or an “intermediate weighting pattern”.

The result figure is shown in Figure 5. The result of hierarchical Bayesian estimations showed that the posterior mean/median and 95% creditable intervals of the group-level weight value had distributions of greater than 0.67, which means that the medial weighting pattern was dominant across participants. In the majority of participants, the posterior mean/median and 95% creditable intervals of individual weights had distributions of greater than 0.67. In only three of 13 participants, posterior means and posterior medians were around 0.5, which represents an intermediate weighting pattern. Fitting results for the data of each participant are described in the Supplementary Results (Supplementary Table 2 and Supplementary Figure 2).

### Comparing the magnitudes of the posterior displacements of the center of pressure between single- and dual-target conditions

**FIGURE 6:**
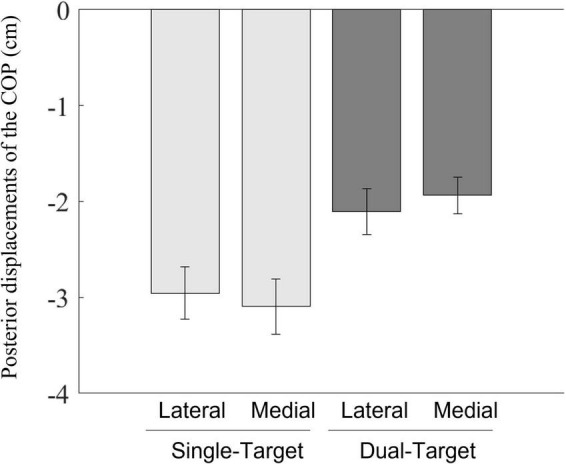
Mean posterior displacements of the COP between the position at the onset of the COP movements and the position at the initial posterior peak of the COP movements toward the swing foot side. Light gray bars represent average values in the single-target condition, and dark gray bars represent average values in the dual-target condition. Error bars represent standard errors of the mean.

The result figure is shown in Figure 6. A two-way ANOVA showed a significant main effect of the number of targets [*F*_(1,12)_ = 31.47, *p* < 0.001, $\eta_{\text{p}}^{2}$ = 0.72]. Posterior displacements of the COP were smaller in the dual-target condition than those in the single-target condition. The main effects of the stepping side and interactions between the number of targets and the stepping side were not significant [*F*_(1,12)_ < 1.0, *p* < 0.832, $\eta_{\text{p}}^{2}$ < 0.01; *F*_(1,12)_ = 4.37, *p* = 0.059, $\eta_{\text{p}}^{2}$ = 0.27, respectively].

### Margin of stability at foot contact

**FIGURE 7:**
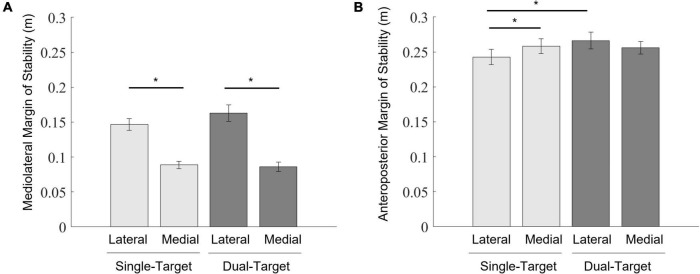
Mean values of the mediolateral MOS **(A)** and the anteroposterior MOS **(B)** at foot contact. Light gray bars represent average values in the single-target condition, and dark gray bars represent average values in the dual-target condition. Error bars represent standard errors of the mean. An asterisk (*) indicates a significant difference in group means based on a *post hoc* analysis (*p* < 0.05).

According to the MOS_ML_ (Figure 7A), a two-way ANOVA showed a significant main effect of the stepping side [*F*_(1,12)_ = 165.46, *p* < 0.001, $\eta_{\text{p}}^{2}$ = 0.93]. The interaction was also significant [*F*_(1,12)_ = 6.43, *p* = 0.026, $\eta_{\text{p}}^{2}$ = 0.35]. Simple main effects of the interaction between two factors revealed that the mediolateral MOS reduction in the single-medial condition was more significant than that in the single-lateral condition [*F*_(1,12)_ = 144.75, *p* < 0.001, $\eta_{\text{p}}^{2}$ = 0.92]. Additionally, the MOS_ML_ reduction in the dual-medial condition was also more significant than that in the dual-lateral condition [*F*_(1,12)_ = 99.28, *p* < 0.001, $\eta_{\text{p}}^{2}$ = 0.89]. There were no other significant effects. In summary, stepping on the medial target led to a greater reduction in the MOS_ML_ than did stepping on the lateral target.

According to the MOS_AP_ (Figure 7B), a two-way ANOVA showed that only interactions were significant [*F*_(1,12)_ = 17.43, *p* = 0.001, $\eta_{\text{p}}^{2}$ = 0.59]. Simple main effects of the interaction between two factors revealed that the MOS_AP_ was increased in the single-medial condition more than in the single-lateral condition [*F*_(1,12)_ = 12.28, *p* = 0.004, $\eta_{\text{p}}^{2}$ = 0.51]. Additionally, the MOS_AP_ was larger in the dual-lateral condition than in the single-lateral condition [*F*_(1,12)_ = 7.70, *p* = 0.017, $\eta_{\text{p}}^{2}$ = 0.39]. There were no other significant effects. In summary, the MOS_AP_ was not reduced when taking a step onto a target in the dual-target condition.

### Movement time and its relationships with the anteroposterior displacements of the center of pressure

The result figure is shown in Supplementary Figure 3. A two-way ANOVA showed the effect of the number of targets, and the interactions were significant [*F*_(1,12)_ = 61.62, *p* < 0.001, *$\eta_{\text{p}}^{2}$* = 0.84; *F*_(1,12)_ = 58.70, *p* < 0.001, *$\eta_{\text{p}}^{2}$* = 0.83, respectively]. Simple main effects of the interaction between two factors revealed that the movement time was shorter in the single-target condition than in the dual-target condition when stepping on the lateral or medial side [*F*_(1,12)_ = 69.29, *p* < 0.001, *$\eta_{\text{p}}^{2}$* = 0.85; *F*_(1,12)_ = 49.72, *p* < 0.001, *$\eta_{\text{p}}^{2}$* = 0.81, respectively]. Additionally, the movement time was shorter in the single-lateral condition than in the single-medial condition, whereas it was longer in the dual-lateral condition than in the dual-medial condition [*F*_(1,12)_ = 34.15, *p* < 0.001, *$\eta_{\text{p}}^{2}$* = 0.74; *F*_(1,12)_ = 15.13, *p* = 0.002, *$\eta_{\text{p}}^{2}$* = 0.56, respectively].

Results of a linear regression fitted to the data of each participant showed that the effect of the magnitude of the posterior COP displacement was significant for 10 of 13 participants (Supplementary Figure 4). According to these participants, the movement time was longer when the magnitude of the posterior COP displacement was smaller.

## Discussion

As hypothesized, lateral displacements of the COP with two potential targets were similar to those when a single target existed on the individual’s medial side. Hierarchical Bayesian estimations of individual strategies showed that medial weighting patterns of the COP were more dominant than intermediate weighting patterns among participants. Only three of 13 participants showed intermediate weighting patterns. Additionally, posterior displacements of the COP with two potential targets became smaller than those with a single target. These results suggest that, when an action involves maintaining upright balance, the action cost for maintaining postural stability is likely to be considered dominantly for planning an anticipatory action.

The results of the lateral COP displacements indicated that the mediolateral components of posture adjustments during the pre-step phase were scaled for stepping onto the individual’s medial side. In consideration of another result that the MOS_ML_ became more destabilized when stepping onto the medial target than when stepping onto the lateral target, these postural adjustments may reflect the anticipatory compensatory strategy to avoid balance disturbances in the mediolateral direction. A similar strategy was reported in other actions performed while standing (Aimola et al., 2011; Xie and Wang, 2019). When there were three objects with different weights and individuals were about to lift one of the objects without knowing the object’s weight, the COP displacements were comparable with those when lifting the heaviest object when instructed about its weight (Aimola et al., 2011). When catching one of three loads with different weights, the COP displacements before catching the object of unknown weight were comparable with those before catching the heaviest object (Xie and Wang, 2019). These anticipatory postural adjustments have been considered to be planning for the worst-case scenario when the weight of an object was uncertain (Eckerle et al., 2012; Xie and Wang, 2019). In line with these studies, the present findings suggest that the brain selects a medial weighting pattern as a predictive compensatory strategy based on the action cost for maintaining postural stability to avoid potential perturbations of balance when stepping onto competing potential targets.

Alternatively, the results of the lateral COP displacements might also be explained in line with energetic efficiency. A previous study has shown that the brain implements an action that minimizes efforts to correct actions after the true target is revealed (Nashed et al., 2017). In the current task settings, participants were often required to adjust the trajectories of their foot and body toward the true target side after leaving the foot on the ground. During such a swing phase, the body falls toward the swing-foot side due to gravity. In this situation, correcting the body toward the lateral side is effortless, but large torques are required to correct the body toward the medial side if needed (Day and Bancroft, 2018). If their bodies are accelerated toward the stance-foot side by shifting the COP largely toward the swing-foot side during the pre-step phase, individuals can step onto the medial target with relatively little torque generation. Considering this, we cannot rule out the possibility that large displacements of the COP toward the swing-foot side in the dual-target condition showed that the brain placed emphasis on energetic efficiencies for correcting stepping actions during a swing phase, rather than postural instabilities. Future studies need to address which explanation would be more suitable—action cost for maintaining balance or energetic efficiency—for the phenomenon of lateral displacements of the COP in preparation for stepping on one of two potential targets.

For three of 13 participants, lateral displacements of the COP were scaled at intermediate locations between those for stepping onto medial or lateral targets. As a reason why the intermediate weighting pattern was selected in the present task, the potential threat to balance disturbances might be less high even when rapid adjustment of the swing foot medially occurred. In our current task settings, the lateral and medial targets were located 10 cm apart from the center target position. Balance disturbances from stepping onto a medial target may have not been sufficient to cause the potential threat to balance disturbances. Participants may have considered that they would be able to correct the swing foot medially without destabilization, even with displacing the COP at an intermediate location. For that reason, tolerances for potential perturbations of balance in accordance with the possible options might affect which strategy is used in the sensorimotor system, as suggested in previous studies using an object-lifting task (Brooks and Thaler, 2017; Cashaback et al., 2017).

In our study, dynamic postural stabilities when landing the swing foot on the target were investigated with the average data of the MOS across trials, showing that stepping onto the medial target became more destabilized. Recently, Kazanski et al. (2021) suggested that an average of the MOS may not be insufficient for evaluating the potential risks of instability (Kazanski et al., 2021). Instead, they proposed that the possibility of instability (POI) that predicts the likelihood of becoming unstable on any future step is more plausible. For the calculation of POI, the mediolateral Margin of Stability (MoS_ML_) smaller than zero indicates instability. In our present study, there were less trials in which the MOS_ML_ was smaller than zero across all participants even when stepping onto the medial target. This suggests that participants in our present study may not have experienced relatively large disturbances of balance.

Posterior displacements of the COP became smaller with two potential targets than with a single target (Figure 6). Additionally, the movement time increased significantly in the dual-target condition (Supplementary Figure 3). For 10 of 13 participants, the movement time increased when the posterior shift of the COP was small (Supplementary Figure 4). This indicates that participants executed their initial actions and completed the task slowly when there were multiple potential targets. These behaviors are consistent with reaching behaviors that reduce the reach speed when the location of the true target is uncertain (Hudson et al., 2007; Onagawa et al., 2022). Onagawa et al. (2022) suggested a smaller initial reaching speed may be selected to increase performance accuracy. For stepping actions, reducing the posterior COP shift contributes to decreasing the velocity of the body for forward progression. These adjustments create extra time to determine which option would be best among potential options and, also, to adjust the swing foot after the true target becomes identified. Therefore, it is possible that the anteroposterior components of the postural adjustments may be organized to accomplish the stepping task successfully.

With regard to how the brain deals with a task with multiple potential options, the motor averaging hypothesis proposed that the brain prepares for multiple potential options and executes an action averaged from these single plans (Chapman et al., 2010; Stewart et al., 2014; Enachescu et al., 2021). Alternately, the motor optimization hypothesis proposed that the brain plans a single action optimized for action costs and task performances (Haith et al., 2015; Wong and Haith, 2017; Alhussein and Smith, 2021). Our results were consistent with the motor optimization hypothesis. In our results, the mediolateral components of the postural adjustments were organized effectively to step onto a medial target. Additionally, the anteroposterior components of the postural adjustments were scaled smaller with two potential targets than with a single target. Based on these findings, we considered that, when two potential targets are located on the medial or lateral sides of the stepping foot, individuals plan an anticipatory action to avoid destabilization with an effort to create extra time to adjust the swing foot toward the true target.

### Limitations

The limitations of the present study were as follows. First, we only set up a situation in which two potential targets were cued as the true target with equal occurrence rates (medial: lateral = 0.5: 0.5). Therefore, the answer to the question is limited as to whether the rule of predominantly considering the action cost for maintaining postural stability would be consistent for other probabilistic situations in which either of two potential targets is selected more frequently (e.g., medial: lateral = 0.2: 0.8).

Second, the sample used for statistical analysis was small, and it was not justified through *a priori* power analysis. We cannot rule out the possibility that no significant differences between the mean of the lateral COP displacements in the dual-target condition and that of the single-medial condition may have been due to the small sample size (in our result, *post hoc* power = 0.31). It is possible that more participants are needed to test the hypothesis that the lateral COP displacements in the dual-target condition were similar to those in the single-medial condition (the medial weighting strategy). Testing with a larger sample is necessary to validate the conclusion of the study with more certainty.

Third, the valid data used for the hierarchical Bayesian estimation was small for some participants (Supplementary Table 1). Although we used the Bayesian modeling method to avoid misestimations from the small data, the estimated parameters from small datasets may not have been sufficiently accurate. Indeed, 95% of creditable intervals of weight values for some participants were widely distributed (Figure 5 and Supplementary Table 2). A larger number of trials are necessary to estimate the individual strategies more accurately.

Fourth, the generalizability of our results using a stepping task remains unknown. In the case of taking a single step, the COM starts from a stationary state and has relatively low momentum. Meanwhile, in the case of walking, the COM moves consistently and has large momentum in both the anteroposterior and mediolateral directions. Therefore, it is possible that, when an individual intended to step onto a target while walking, the COM trajectory and the COM momentum during a pre-step phase dominantly affect planning of a stepping action. Indeed, deviations from the target location to the direction of the COM momentum at foot lift affect the magnitude of the foot correction (Barton et al., 2019). For these reasons, a strategy for controlling the body and foot while walking might be more flexibly selected based on not only the action costs for maintaining postural stability but also the COM movements to the present time point.

## Conclusion

Our results suggest that individuals prepare the mediolateral components of their postural adjustments for stepping toward the medial side effectively to avoid upcoming postural disturbances. Additionally, the anteroposterior components of the postural adjustments became smaller, possibly to create extra time to determine the true target and to adjust the swing foot. Based on our results, we concluded that the action costs for maintaining postural stability were considered dominantly for planning an anticipatory action to accomplish a stepping task successfully while ensuring upright balance.

## Data availability statement

The original contributions presented in this study are included in the article/Supplementary material, further inquiries can be directed to the corresponding author.

## Ethics statement

The studies involving human participants were reviewed and approved by the Ethics Committee of Tokyo Metropolitan University. The patients/participants provided their written informed consent to participate in this study.

## Author contributions

RW: conceptualization, methodology, data curation, formal analysis, and writing of the original draft. TH: conceptualization, methodology, and writing – review and editing. Both authors contributed to the article and approved the submitted version.
