# Supplementary material for: Anticipatory action planning for stepping onto competing potential targets
Source: Front Hum Neurosci. 2022 Aug 22;16:875249. doi: 10.3389/fnhum.2022.875249 (PMC9441706; doi:10.3389/fnhum.2022.875249)
Supplement: Supplementary file 1 [file Data_Sheet_1.docx]

**Supplementary Materials**

|  | Single – Center | Single – Lateral | Single – Medial | Dual – Lateral | Dual – Medial | Excluded data of　 dual-target condition |
| --- | --- | --- | --- | --- | --- | --- |
| ID1 | 38 | 42 | 26 | 31 | 32 | 33 |
| ID2 | 38 | 42 | 30 | 7 | 3 | 86 |
| ID3 | 36 | 39 | 23 | 24 | 20 | 52 |
| ID4 | 44 | 44 | 42 | 37 | 40 | 19 |
| ID5 | 32 | 38 | 36 | 38 | 35 | 23 |
| ID6 | 22 | 13 | 14 | 23 | 25 | 48 |
| ID7 | 41 | 37 | 33 | 37 | 35 | 24 |
| ID8 | 25 | 25 | 18 | 19 | 19 | 58 |
| ID9 | 11 | 15 | 17 | 11 | 10 | 75 |
| ID10 | 12 | 18 | 14 | 17 | 15 | 64 |
| ID11 | 39 | 39 | 25 | 31 | 34 | 31 |
| ID12 | 28 | 29 | 22 | 22 | 25 | 49 |
| ID13 | 35 | 38 | 25 | 34 | 34 | 28 |
| Average | 30.8 | 32.2 | 25.0 | 25.5 | 25.2 | 45.4 |
| SD | 10.2 | 10.5 | 8.2 | 9.8 | 10.8 | 20.4 |

**Supplementary Table 1.** Valid data of each stepping condition and exclude data of the dual-target condition for each participant

| Estimated theta[ID] | Posterior　mean | Standard error　of posterior mean | Standard deviation | Creditable interval 95% percentile [lower upper] | | $\hat{R}$ |
| --- | --- | --- | --- | --- | --- | --- |
| θ | 0.983 | 0.00026 | 0.047 | 0.888 | 0.985 | 1.000 |
| θ[1] | 0.996 | 0.00005 | 0.010 | 0.966 | 0.997 | 1.000 |
| θ[2] | 0.549 | 0.00170 | 0.314 | 0.000 | 0.321 | 1.000 |
| θ[3] | 0.572 | 0.00030 | 0.083 | 0.411 | 0.517 | 1.000 |
| θ[4] | 0.999 | 0.00002 | 0.004 | 0.988 | 0.999 | 1.000 |
| θ[5] | 0.969 | 0.00033 | 0.055 | 0.807 | 0.961 | 1.000 |
| θ[6] | 0.849 | 0.00063 | 0.077 | 0.716 | 0.795 | 1.000 |
| θ[7] | 0.474 | 0.00025 | 0.069 | 0.338 | 0.428 | 1.000 |
| θ[8] | 0.997 | 0.00003 | 0.007 | 0.978 | 0.998 | 1.000 |
| θ[9] | 0.804 | 0.00143 | 0.159 | 0.503 | 0.680 | 1.001 |
| θ[10] | 0.996 | 0.00005 | 0.011 | 0.963 | 0.997 | 1.000 |
| θ[11] | 0.995 | 0.00006 | 0.012 | 0.957 | 0.996 | 1.000 |
| θ[12] | 0.976 | 0.00026 | 0.049 | 0.825 | 0.977 | 1.000 |
| θ[13] | 0.991 | 0.00010 | 0.020 | 0.927 | 0.994 | 1.000 |

**Supplementary Table 2.** Parameters with Hierarchical Bayesian estimates of the model fitting to the pooled data from all participants. θ represents a group weight between the policies of medial stepping and lateral stepping, which is an group-level parameter. θ[ID] represents an individual weight between the policies of medial stepping and lateral stepping, which is an individual-level parameter. A convergence check was executed based on R-hat diagnostic values ($\hat{R}$). The values among all parameters were below 1.1.


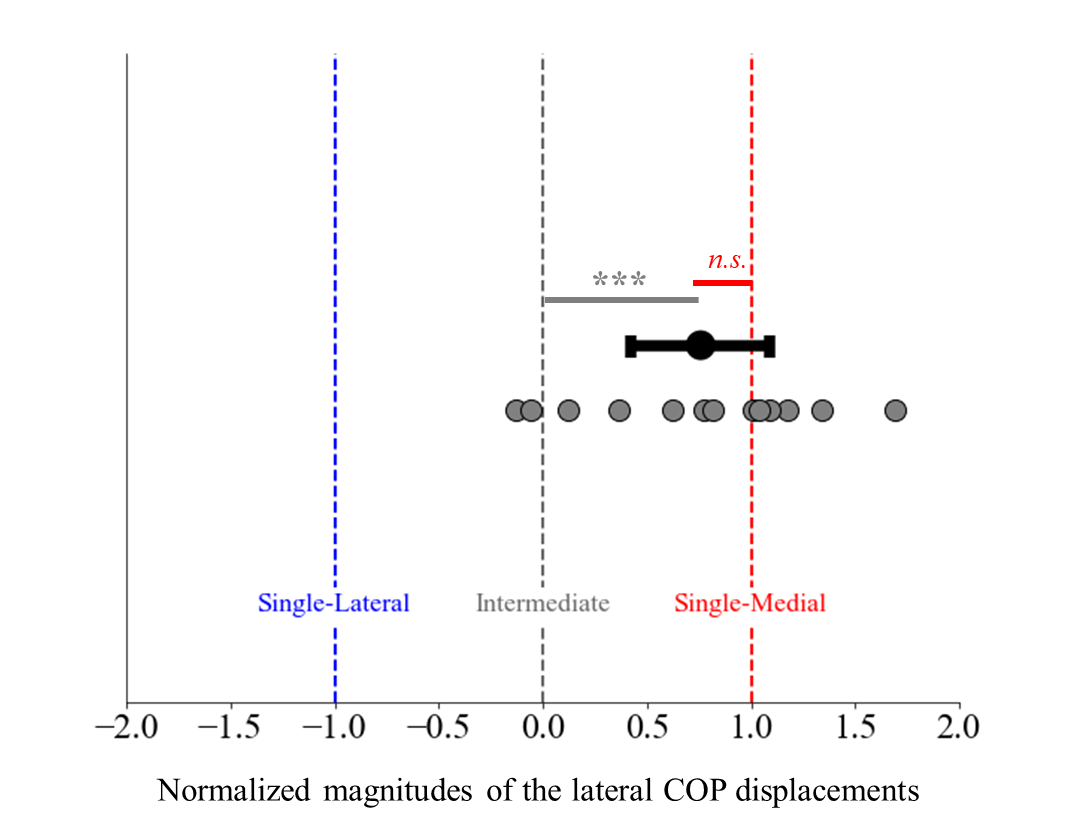


**Supplementary Figure 1.** Mean values of the normalized magnitudes of the lateral COP displacements. The round dot filled in black represents a mean of the normalized magnitudes of the lateral COP displacements averaged across all participants (*n* = 13). An error bar represents a 95 % confidential interval of the mean. Round Dots filled in gray represent each participant’s mean. An asterisk (***) indicates a significant difference of group means based on a one-sample t test comparing with mean = 0 (*p* < .001). *n.s.* indicates a nonsignificant difference of group means comparing with mean = 1 (*p* = .142).


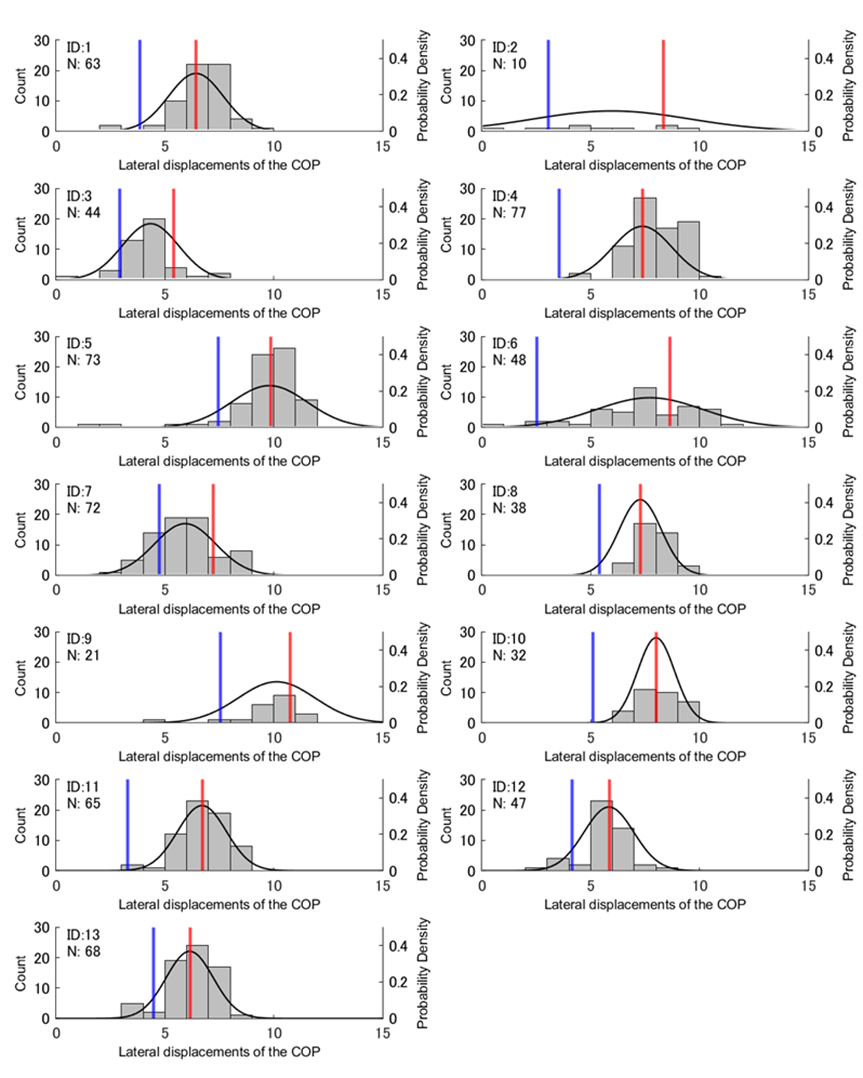


**Supplementary Figure 2.** Histograms of the lateral displacements of the COP for each participant in the dual-target condition and Gaussian functions drawn using estimated parameters. Each panel represents each individual participant’s data. Gray histograms represent the distributions of lateral displacements of the COP pooled over the lateral target condition and the medial target condition in the dual-target condition for each participant (bin width: 1 cm). Smooth lines represent Gaussian functions using the individual weight between the policy of medial stepping and lateral stepping for each participant and the standard distribution. A blue and red line represent mean values of the lateral displacements of the COP in the single-lateral and single-medial condition, respectively. We noted that numbers of valid data of participant ID2 were extremely few as compared with those of other participants.


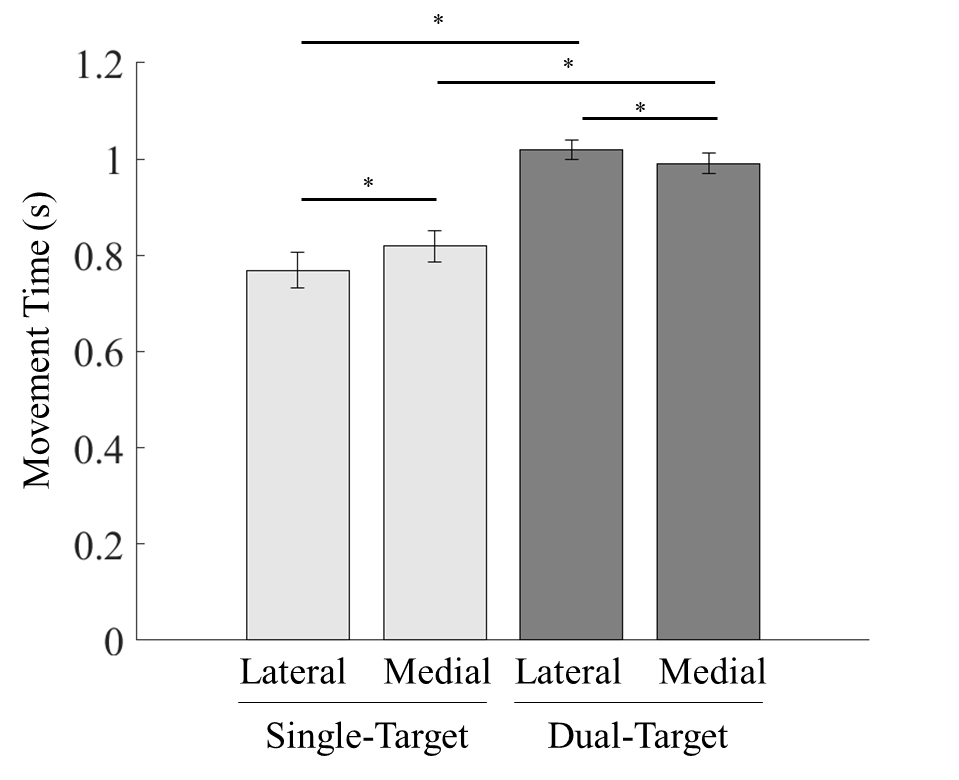


**Supplementary Figure 3.** Mean values of the movement time which indicate the time from the event of the COP onset to the event of foot contact. Light gray bars represent average values in the single-target condition and dark gray bars represent average values in the dual-target condition. Error bars represent standard errors of the mean. An asterisk (*) indicates a significant difference in group means based on a post hoc analysis (*p* < .05).


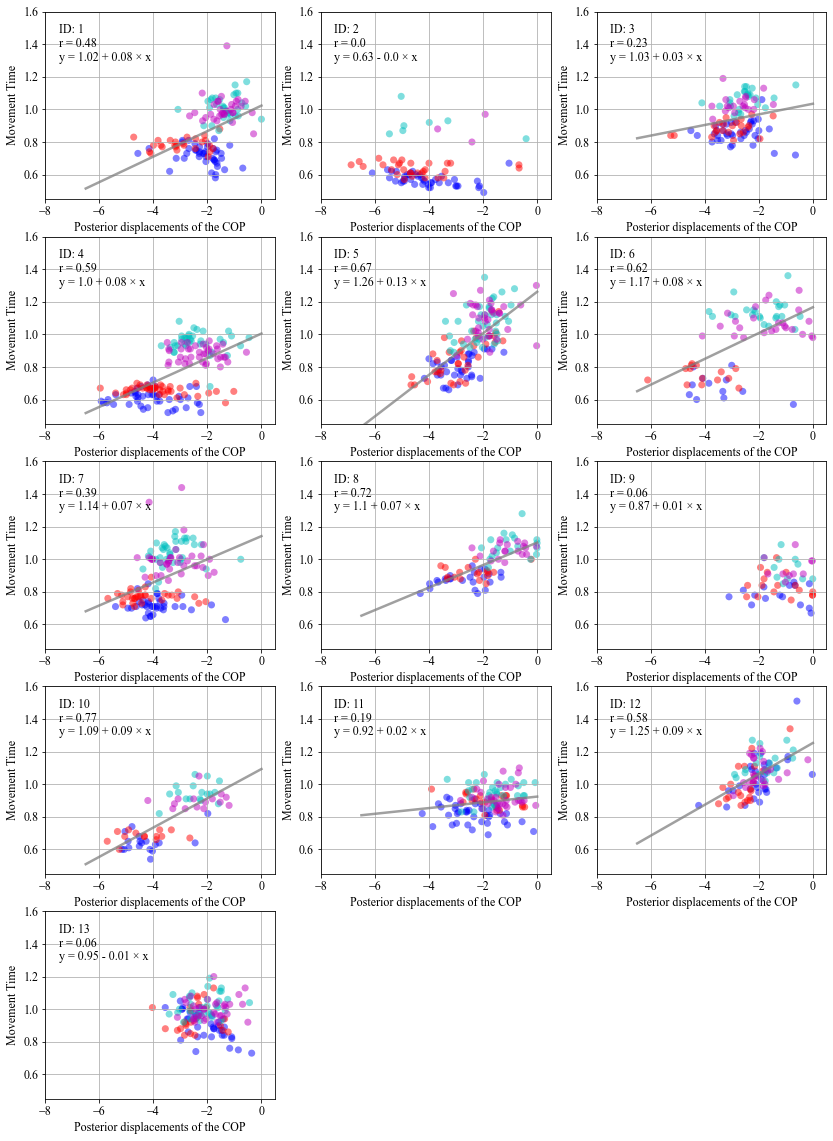


**Supplementary Figure 4.** Data plots illustrating the relationship between the posterior displacements of the COP and the movement times for each participant. Each panel represents each participant’s data. Round dots represent the posterior displacements of the COP and the movement time for each trial in the single-lateral (blue), single-medial (red), dual-lateral (cyan) or dual-medial (magenta) condition. A Smooth line represents a linear regression estimated from the data pooled from each trial in the single-lateral condition, the single-medial condition, the dual-lateral condition and the dual-medial condition for each participant. These lines are only showed when a *p* value of the regression coefficient was lower than the threshold of significance (*p* < 0.05).
